# A Meta-Analysis of the Efficacy of Hyaluronic Acid Eye Drops for the Treatment of Dry Eye Syndrome

**DOI:** 10.3390/ijerph18052383

**Published:** 2021-03-01

**Authors:** Yun-Jung Yang, Won-Young Lee, Young-jin Kim, Yeon-pyo Hong

**Affiliations:** 1 Institute of Biomedical Science, Catholic Kwandong University International St. Mary’s Hospital, Incheon 22711, Korea; yangyj@ish.ac.kr; 2 Department of Preventive Medicine, College of Medicine, Chung-Ang University, Seoul 06974, Korea; wylee@cau.ac.kr; 3 Executive Director of the Korean Contact Lens Study Society, Seoul 07345, Korea; 0zeeny@gmail.com

**Keywords:** dry eye, hyaluronic acid, meta-analysis

## Abstract

Hyaluronic acid (HA) is commonly used for treating dry eye syndrome (DES). This meta-analysis was performed to compare the efficacies of HA- and non-HA-based eye drops, including saline and conventional artificial tears (ATs), for the treatment of dry eye disease. Eight databases (PubMed, EMBASE, Cochrane Central Register of Controlled Trials, DBpia, KoreaMed, KMBASE, RISS, KISS) were searched for studies comparing the efficacies of HA- and non-HA-based ATs in patients with DES published up to September 2020. Two independent reviewers assessed the quality and extracted the relevant data. The mean differences of Schirmer’s (SH) test scores, tear breakup times (TBUT), corneal fluorescein staining scores (Oxford scale, 0–4), and ocular surface disease indexes were calculated. The standard mean difference and 95% confidence interval were calculated using a random effect model. Nineteen studies, including 2078 cases, were included. HA eye drops significantly improved tear production compared with non-HA-based eye drops (standard mean difference (SMD) 0.18; 95% confidence interval (CI) 0.03, 0.33). In a subgroup analysis, the SH test scores and TBUT values after using HA significantly increased compared to those measured after using saline (SMD 0.27; 95% CI 0.05, 0.49 and SMD 0.28; 95% CI 0.03, 0.52, respectively). Based on these results, HA eye drops may be superior to non-HA eye drops including normal saline and ATs. Further research is needed to assess the efficacies stratified by age, treatment duration, the severity of dry eye, and optimal dosages.

## 1. Introduction

Dry eye syndrome (DES) results from insufficient tear production or excessive evaporation of tears, and is associated with symptoms such as dry eye surface, discomfort, visual impairment, and aching [1]. It also leads to an increase in the osmolality of the tear film and inflammation of the ocular surface. The prevalence of DES is estimated as 5–30% in people older than 50 years [2].

Although DES is a very common disease in adults [3,4], its diagnostic and treatment assessment methods have not yet been standardized. To diagnose patient symptoms, a self-reported questionnaire such as the ocular surface disease index (OSDI) is used. In addition, various clinical tests, including the Schirmer (SH) test, tear breakup time (TBUT), corneal and conjunctival staining, tear meniscus height, tear osmolality, and tear lysozyme analysis, are conducted by clinicians [5]. 

Several treatment options are available to patients with DES depending on the severity of their symptoms. For treating dry eye, tear replacement products or punctal plugs are used to restore the original homeostasis of the ocular surface and tear film. Recently, several pharmacologic agents have been used to stimulate tear production [6]. Tear replacement with numerous kinds of lubricants is used to improve ocular surface discomfort. Products called artificial tears (ATs), including hyaluronic acid (HA), polyacrylic acid, carboxymethyl cellulose (CMC), dextran, HP-guar, hydroxypropyl methyl cellulose, polyvinyl alcohol, polyvinylpyrrolidone, and polyethylene glycol, are available [6]. Because these products lack the biologically active ingredients found in natural tears [7,8], they may be used in combination with other supplements to enhance lubrication and lengthen the time they last on the ocular surface before evaporation. 

HA or sodium hyaluronate is a glycosaminoglycan disaccharide linear biopolymer consisting of repeated alternating sequences of N-acetyl-glucosamine and glucuronate [9]. The topical application of HA has been used to increase the secretion of water and mucin on the ocular surface since the early 1990s [10]. The beneficial effects of various concentrations of HA eye drops on the ocular surface, tear film stability, and dry eye symptoms have been reported in humans [11,12,13,14] and in animal models [15]. However, some studies have been reported that tear supplement with ATs other than HA eye drops significantly improved dry eye signs and symptoms and relieved inflammation [16,17,18,19,20]. It seems that topical preparation of HA has been provided a considerable improvement of subjective and objective outcomes in patients with DES, while there is controversy regarding the efficacy of HA-only eye drops treatment. 

Some systematic reviews and meta-analyses performed a pooling analysis of data to compare the efficacies of HA- and non-HA-based eye drops for treating DES [21,22,23]. The results of objective indicators, including TBUT and remission rate, did not show significance [22,23]. In another study, the significant difference of pre- and posttreatment on SH test and TBUT showed 0.238 mm and 0.566 s, respectively [21]. The authors insisted that these differences might be not enough to reflect the clinical significance but are truly comparable. Therefore, there is a need for more reliable results on the effectiveness of HA eye drops for relief of DES.

This study was performed to compare the efficacies of HA-only eye drops and non-HA-based eye drops using common objective and subjective outcomes. Non-HA-based eye drops were classified into saline and ATs. This is because some studies have used saline as comparator in DES treatment but have not used any lubricants. The objective outcomes were SH test score, TBUT, and corneal fluorescein staining score, and the subjective outcome was OSDI.

## 2. Materials and Methods

### 2.1. Literature Search

PubMed, the Cochrane Central Register of Controlled Trials, EMBASE, DBpia, KoreaMed, KMBASE, RISS, and KISS databases were searched for studies published up to September 2020. The search terms were (“dry eye” or “keratoconjunctivitis sicca” or “Sjogren’s syndrome” or “xerophthalmia”) and (“hyaluronic acid” or “hyaluronan”). There were no restrictions on sources or languages.

### 2.2. Inclusion and Exclusion Criteria

The inclusion criteria were as follows: (1) Type of studies: Randomized controlled trials (RCT); (2) Type of participants: Patients with DES, not restrictions for age, gender, or race; (3) Type of interventions: Topical HA-only eye drops with different concentrations; (4) Type of comparisons: Non-HA-based eye drops, including ATs and normal saline; (5) Type of outcomes: At least 1 outcome of SH test, TBUT, corneal fluorescein staining scores (Oxford score scale, 0–5), and OSDI; and (6) Follow-up duration: At least 7 days after the initiation of treatment with eye drops. 

The exclusion criteria were as follows: (1) Not RCT, i.e., observational studies, self-controlled studies, clinical trials without contrast, reviews, and letters; (2) Abstracts and conference proceedings; (3) Previously conducted cataracts or ocular surgery; (4) Previously used eye drops for therapeutic purposes (e.g., glaucoma) or contact lens wear; (5) First follow-up after 5 or more weeks; and (6) Not published in English or Korean. 

### 2.3. Study Selection 

The abstracts and titles of all selected studies were independently reviewed by 2 authors. Unrelated articles were immediately excluded. The full texts of the remaining articles were reviewed. Articles that met the inclusion criteria were finally selected, and the data of updated publications involving the same cohort of cases were extracted synthetically.

### 2.4. Data Extraction

Relevant information and data were extracted from the selected studies, including the first author’s name, year of publication, country of study, general characteristics of participants, disease severity, randomization, masking, follow-up duration, % of HA, and control eye drop used (Table 1). In crossover studies with more than 1 treatment period for the same eye drop, the first trial results were extracted for analysis.

To ensure homogeneity, the data obtained within 1 to 5 weeks after the initiation of treatment with eye drops were extracted for analysis. This was determined by referring to a previous study [21]. When the studies reported more than 1 non-HA-based eye drop treatment, each datum was individually included. When the studies separately reported the scores for the left and the right eyes, the results of the right eye were included to avoid analytic errors.

### 2.5. Quality Assessment

The quality of studies included in the meta-analysis was assessed using the Cochrane Risk of Bias Tool [24]. Two authors independently assessed the risk of selection bias, performance bias, attribution bias, reporting biases, and other bias of each study. Disagreements were resolved by the 2 authors by discussion or consultation with the third author. The risks of bias for 19 studies were classified as low, high, or unclear.

### 2.6. Statistical Analysis

A meta-analysis was performed using STATA/MP v16 (StataCorp, College Station, TX, USA). For the quantitative analysis, the changes in the means and standard deviations of the SH test score, TBUT, corneal staining score, and OSDI from baseline to follow-up were used. When the change was not reported by a study, it was calculated as follows [24]:

$$Mean_{change}=Mean_{endpoint}-Mean_{baseline}$$

$$SD_{change}=\sqrt{{\left(SD_{baseline}\right)}^{2}+{\left(SD_{endpoint}\right)}^{2}-2\times r\times SD_{baseline}\times SD_{endpoint}}$$

where *r* represents the correlation coefficient. We took *r* = 0.5 in this study.

The standard mean difference (SMD) and 95% confidence interval (CI) were calculated for outcomes. A planned subgroup analysis was performed based on the components of non-HA-based eye drops, including normal saline and ATs. Heterogeneity was assessed using the Chi-squared test and the I^2^ statistic. When heterogeneity was low (I^2^ < 50%, *p* > 0.1), a fixed effect model was used. When significant heterogeneity (I^2^ > 50%, *p* < 0.1) was detected, a sensitivity analysis was performed. If heterogeneity could not be eliminated, a random effect model was used. 

Publication bias was evaluated using the funnel plot and the Egger linear regression test. A *p*-value of less than 0.05 was considered to indicate statistical significance.

## 3. Results

### 3.1. Literature Retrieval Results

**Figure 1:**
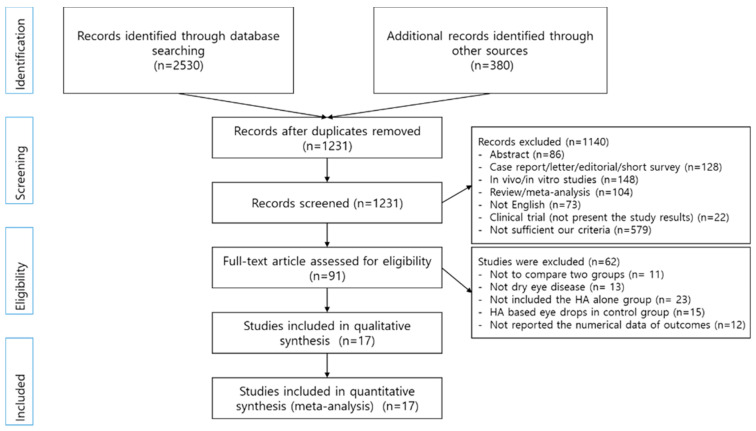
Study selection process.

Initially, 2910 articles were searched, and 1679 duplicate studies were removed. Among the remaining studies, 1140 articles were excluded based on their titles and abstracts (Figure 1). Full texts and data were reviewed for 91 studies. Of these, 17 studies contained results of at least 1 on the SH test, TBUT, corneal staining score, and OSDI [11,12,16,18,19,20,25,26,27,28,29,30,31,32,33,34,35].

### 3.2. Study Characteristics

The 17 studies finally included were published between 1988 and 2018. Of these, 13 were conducted in Europe [11,12,16,18,19,25,26,27,28,29,30,31,34], 2 in Asia [20,33], 1 in the United States [35], and 1 in the Canada [32] (Table 1). Based on the type of study, 12 studies were parallel, and 5 studies were crossover. All parallel and crossover studies used randomization. Fifteen studies described a single or double masking, and one study was open label. The remaining study did not indicate whether masking was carried out. The included subjects were patients with dry eye of various severities, including mild, mild to moderate, moderate, moderated to severe, and moderately to severe. Seven out of a total of 17 documents did not describe the severity. The follow-up duration ranged from 14 to 90 days. There were 627 cases for the HA eye drops group and 712 cases for the non-HA-based eye drops group. One study reported the number of eyes instead of the number of subjects [16]. The average age was mostly 50–60 years. One of the other two cases was 38 years old and the other one was 72 years old. The number of women were relatively higher than man. Six studies used a 0.1% concentration of HA, and the remaining 11 studies used 0.15–0.4% HA. The most common component in the non-HA based eye drops was methylcellulose (*n* = 6). Six studies used emulsion, polyvinyl alcohol, and carbomer. Saline was used in four studies.

### 3.3. Methodological Quality Assessment

A quality assessment was performed for 17 studies (Table 2). Although all studies were performed with randomization, only three studies described the randomization method and allocation concealment [20,25,31]. Most of the studies did not describe the randomization method, and selection bias could not be determined. Because 15 of 17 studies were investigator-/assessor-blinded [11,12,16,19,20,25,26,27,29,30,31,33,34,35], the detection bias was low. In addition, the blinding of participants was performed in 11 studies [11,12,19,25,26,27,28,31,34,35]. All studies were assessed as having low risks of attrition bias and reporting bias. Besides two studies showing significantly different mean ages at baseline, the studies were classified unclear of other bias [16,35].

### 3.4. Quantitative Analysis

#### 3.4.1. Schirmer’s (SH) Test

**Figure 2:**
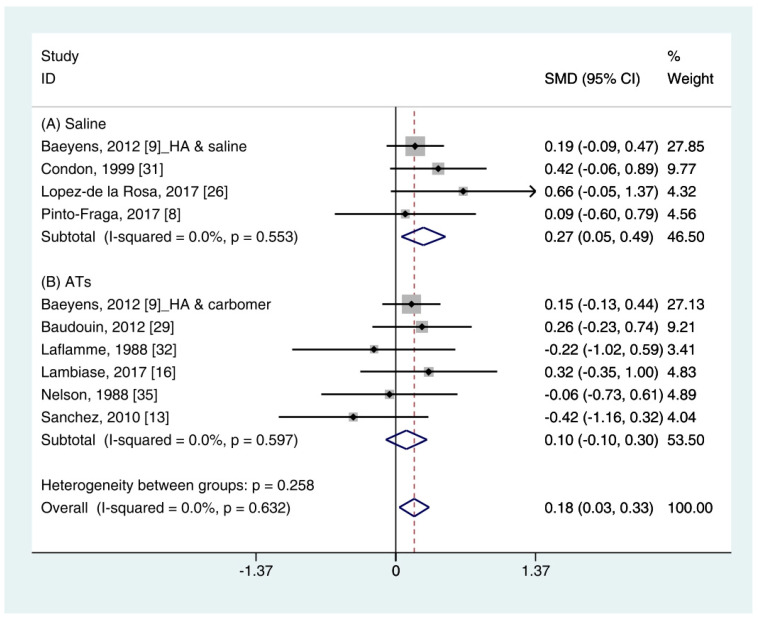
Comparison of the changes on SH test scores (mm/5 min) in HA and non-HA groups using the fixed effect model. The non-HA group was classified into (A) saline and (B) ATs depending on whether lubricant was included. Subgroup analysis was performed between HA and (A) saline and between HA and (B) ATs. SH test: Schirmer’s test; HA: Hyaluronic acid; ATs: Artificial tears; SMD: Standardized Mean Difference; CI: Confidence interval.

The quantitative data of the SH test (*n* = 10) were available from nine studies [11,12,16,19,26,29,31,32,35]. A pooled total of 362 cases was randomly allocated to the HA group, and 348 cases were assigned to the non-HA group. A pooled analysis showed that the HA eye drops significantly increased tear production compared with the non-HA group (SMD 0.18; 95% CI 0.03, 0.33), with low heterogeneity (I^2^ = 0.0%, *p* = 0.632) (Figure 2). 

In the subgroup analysis, the HA group significantly improved the SH test scores compared with the saline group (SMD 0.27; 95% CI 0.05, 0.49), with low heterogeneity (I^2^ = 0.0%, *p* = 0.553). The SH test scores between the HA and the ATs group were similar (SMD 0.10; 95% CI −0.10, 0.30), with low heterogeneity (I^2^ = 0.0%, *p* = 0.597). Publication bias was not observed for saline (Egger’s test, t = 0.24, *p* = 0.833) or ATs (Eggers test, t = 1.64, *p* = 0.176).

#### 3.4.2. Tear Break-Up Time (TBUT)

**Figure 3:**
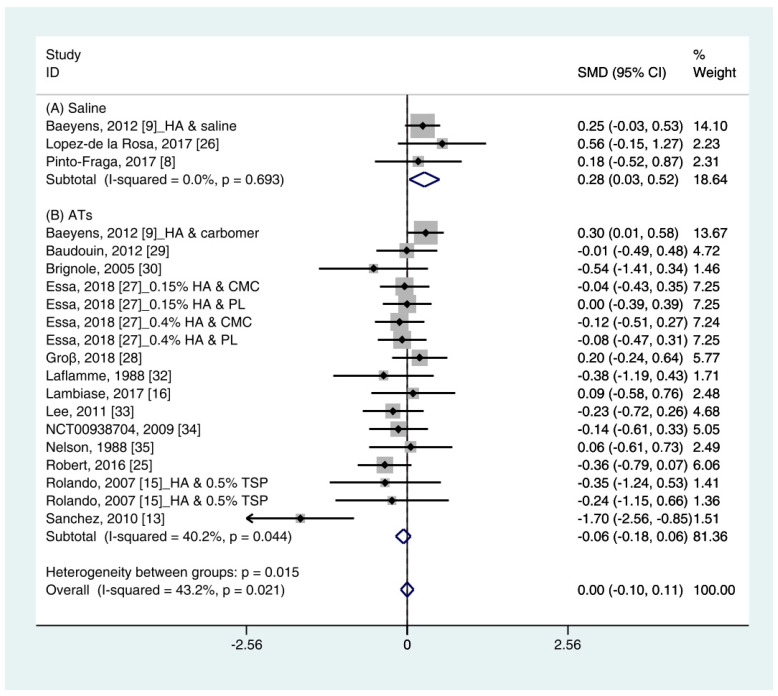
Comparison of the changes on TBUT values (seconds) in HA and non-HA groups using the fixed effect model. The non-HA group was classified into (A) saline and (B) ATs depending on whether lubricant was included. Subgroup analysis was performed between HA and (A) saline and between HA and (B) ATs. TBUT: Tear break-up time; HA: Hyaluronic acid; ATs: Artificial tears; SMD: Standardized Mean Difference; CI: Confidence interval.

Data (*n* = 21) from 15 studies were included in the meta-analysis of the TBUT outcomes [11,12,16,18,19,25,26,27,28,29,30,32,33,34,35]. A pooled total of 707 cases was randomly allocated to the HA group, and 693 cases were randomly allocated to the non-HA group. The mean changes in TBUT were similar between the HA and non-HA group (SMD −0.00; 95% CI −0.10, 0.11), and overall heterogeneity was high (I^2^ = 43.2%, *p* = 0.021) (Figure 3). 

In the subgroup analysis, a significant improvement of tear film stability was observed in the HA group (SMD 0.28; 95% CI 0.03, 0.52), with low heterogeneity (I^2^ = 0.0%, *p* = 0.693) compared with the saline group. The TBUTs between the HA and the ATs group were similar (SMD −0.06; 95% CI −0.18, 0.06), with high heterogeneity (I^2^ = 40.2%, *p* = 0.044). Publication bias was detected in the ATs group (Egger’s test, t = 2.60, *p* = 0.020). After excluding the source of heterogeneity [16], heterogeneity was decreased (I^2^ = 0.0%, *p* = 0.649). However, the HA group showed similar improvement of TBUT compared with the ATs group (SMD −0.03; 95% CI −0.15, 0.09) (Figure S1).

#### 3.4.3. Corneal Fluorescein Staining Score

**Figure 4:**
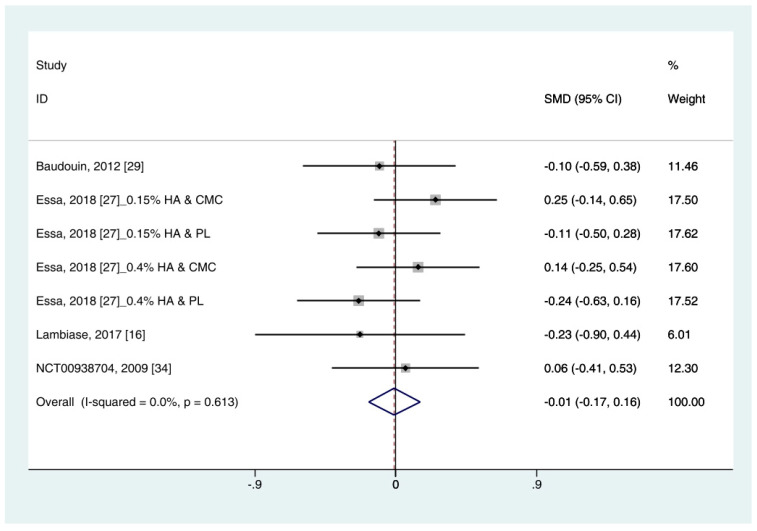
Comparison of the changes on corneal fluorescein staining score (Oxford scale, 0–4) in (HA) and ATs using the fixed effect model. The corneal fluorescein staining score was only extracted from ATs. HA: Hyaluronic acid; ATs: Artificial tears: SMD: Standardized Mean Difference; CI: Confidence interval.

Data on corneal fluorescein staining score (*n* = 7) were obtained from four studies [19,27,29,34]. Pooled data from 286 cases were randomly allocated to the HA group, and data from 272 cases were allocated to the ATs group. The corneal fluorescein staining score was only extracted for ATs. Thus, subgroup analysis was not performed. A similar improvement was observed for HA and ATs (SMD −0.01; 95% CI −0.17, 0.16), with low heterogeneity (I^2^ = 0.0%, *p* = 0.613) (Figure 4). The results did not show a publication bias (Eggers test, t = 0.72, *p* = 0.501).

#### 3.4.4. Ocular Surface Disease Index (OSDI)

**Figure 5:**
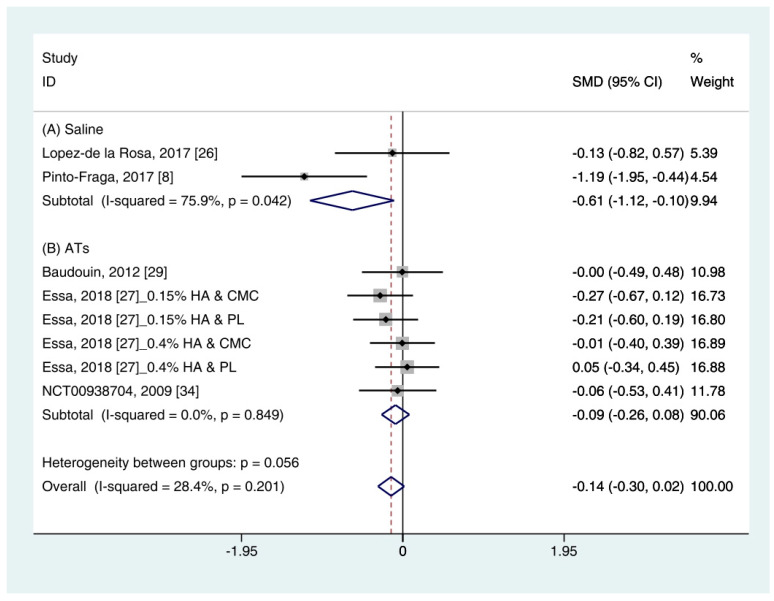
Comparison of the changes on OSDI value in HA and non-HA groups using the fixed effect model. The non-HA group was classified into (A) saline and (B) ATs depending on whether lubricant was included. Subgroup analysis was performed between HA and (A) saline and between HA and (B) ATs. OSDI: Ocular surface disease index; HA: Hyaluronic acid; ATs: Artificial tears; SMD: Standardized Mean Difference; CI: Confidence interval.

Data (*n* = 8) from five studies were included in the meta-analysis of OSDI outcomes [11,26,27,29,34]. A pooled total of 298 cases was randomly allocated to the HA group, and 284 cases were randomly allocated to the non-HA group. Based on the pooled data, the HA group tended to show decreased symptoms of DES compared with the non-HA group. However, the difference was not significant (SMD −0.14; 95% CI −0.30, 0.02) (Figure 5). 

In subgroup analysis, the HA group was significantly decreased the symptoms compared with saline (SMD −0.61; 95% CI −1.12, −0.10), with high heterogeneity (I^2^ = 75.9%, *p* = 0.042). The OSDI score showed similar between the HA and the Ats groups (SMD −0.09; 95% CI −0.26, 0.08), with low heterogeneity (I^2^ = 0.0%, *p* = 0.849). 

The publication bias was detected between the HA and the saline groups. However, there were only two studies in the saline group [11,26]. Thus, the OSDI score between the HA group and the saline group were re-examined using the random effect model. The HA group tended to show more improvement of symptoms compared to the saline group. However, statistical significance was not observed (SMD −0.65; 95% CI −1.69, 0.40) (Figure S2).

## 4. Discussion

The use of HA eye drops has increased in patients with various ocular surface disorders because of its water retention and lubricant properties. In previous studies, the superiority of HA for treating DES was not clearly reported [21,22,23]. Thus, this study aimed to assess the effects of HA eye drops on DES compared with non-HA eye drops, including saline and ATs. For the quantitative analysis, objective tests (e.g., SH test, TBUT, and corneal staining score (Oxford scale, 0–4)) and subjective tests (e.g., OSDI) were used.

A pooled analysis showed that the HA group significantly improved the tear production (based on the SH test) compared with the non-HA group (SMD 0.18; 95% CI 0.03, 0.33) with low heterogeneity (I^2^ = 0.0%, *p* = 0.632). The corneal fluorescein staining scores and TBUT values were similar in the HA- and non-HA group were similar. The HA eye drops tended to decrease the OSDI compared with the non-HA eye drops. However, statistical significance was not observed.

In a subgroup analysis, the HA group significantly increased the tear production (based on the SH test) and tear film stability (based on the TBUT) compared with normal saline (SMD 0.27; 95% CI 0.05, 0.49, and SMD 0.28; 95% CI 0.03, 0.52, respectively). Symptom scores (based on the OSDI) in the HA group significantly decreased compared with the saline group (SMD −0.61; 95% CI −1.12, −0.10) with high heterogeneity (I^2^ = 75.9%, *p* = 0.042). By random effect modeling, the HA group showed similar OSDI score compared with the saline group (SMD −0.65; 95% CI −1.69, 0.40). The mean changes of SH test score, TBUT, corneal fluorescein score, and OSDI score showed similar between the HA and ATs groups.

The SH test, which measures tear production, is a commonly used diagnostic method in ophthalmology [36]. However, the large measurement error and poor reproducibility are considered limitations [37]. In this study, HA eye drops significantly improved the SH test score compared with non-HA eye drops (Figure 2). Because the changes between the HA- and the non-HA groups was small (0.18 mm), it might not have a significant impact on patient symptoms. Individual studies in this study reported that HA- and non-HA eye drops were effective in the treatment of DES. When considering the various conditions of individual studies, it is reasonable that this degree of difference appears. A previous meta-analysis also showed a significant improvement in the SH test score (0.238 mm) after using an HA eye drops compared with a non-HA eye drops [21]. Two studies were included in the previous meta-analysis but not in our study [38,39]. Those studies reported the beneficial effects of HA on the treatment of DES. However, they did not meet the inclusion criteria in this study because one study used HA-based polyethylene glycol [38] and the other study did not provide a standard deviation [39]. Nonetheless, the overall data showed the significant improvement of tear production after HA treatment compared with the non-HA treatment (Figure 2).

Similar to the SH test score, the TBUT after using HA significantly improved than that of saline and similar to that of ATs (Figure 3). In addition, the ATs outcomes showed high heterogeneity. The TBUT is used to assess tear film stability [40]. It measures the elapses time between the end of a complete blink and the appearance of the first break in the tear film [36]. It is widely used in clinical practice because it can relatively easily measure tear production. A previous meta-analysis showed less improvement in TBUT after using an HA compared with a non-HA preparation, but significance was not observed [21,23].

Among previous studies and our study, only one study reported that non-HA preparation was more effective than HA preparation based on TBUT [16]. The study by Sanchez et al. (2010) [16], which appeared to be the cause of heterogeneity in the previous studies, was also detected as an outlier. This study only reported a significant improvement in TBUT after using ATs compared with HA eye drops. They explained that the difference between the ages of the two groups may have resulted in a difference in the efficacy of treatment. After excluding the study, the heterogeneity was reduced, but there were no significant changes in the SMD of HA and ATs (Figure S1).

The corneal fluorescein staining score after using HA eye drops was similar to that of ATs (Figure 4). Sodium fluorescein, rose Bengal, and lissamine green are widely used staining agents for diagnosing ocular disease. There are various grading systems for recording the severities of ocular surface disorders. This study only used the corneal fluorescein staining score based on the Oxford Scheme (scale 0–4). According to the criteria, low heterogeneity was observed. Although the corneal staining scores is considered informative marker for severe DES, patients with mild/moderate DES showed a poor correlation [41]. Thus, it may be poorly associated with subjective symptoms.

In addition to these objective methods, the HA group showed similar improvement of the OSDI scores compared with the non-HA group (Figure 5). However, significance was not observed. OSDI is widely used to assess DES in clinical trials. It measures the frequency of symptoms, environmental conditions, and vision-related quality of life [36]. Although symptoms are scored by the individual, it is known to produce more reliable and reproducible results than other objective tests [42]. Thus, subjective indicators such as OSDI may be important indicators for evaluating response to DES treatment.

This study has several limitations. First, there was a restriction on the data extraction point. Numerous RCTs used blinding or masking to reduce bias. However, it is difficult to maintain blinding or masking throughout studies because of the instillation frequency, chemical properties, and ocular sensation after instilling HA and non-HA ophthalmic solutions. Thus, we selected the data obtained within 5 weeks and after 1 week of administration to extract homogenous data as much as possible. This period was determined based on a previous study [21]. Second, there were various types of ingredients in the non-HA eye drops, including saline, CMC, phospholipid liposome, emulsions, rebamipid, carmellose, lubricin, and tamarind seed polysaccharide. To reduce heterogeneity and increase accuracy, the comparison group was divided into saline-and AT groups. Third, it is not known whether statistical significance implies clinical improvement. Although the statistically significant beneficial effects of HA, compared with saline, were reflected in the SH test scores and TBUTs, it is difficult to conclude that these differences are clinically meaningful for the treatment of DES. Therefore, further trials are needed to determine the clinical relevance of the symptoms of DES and test outcomes.

Nonetheless, this study included a relatively larger sample size than previous studies that have evaluated the effect of HA. Both objective and subjective indicators were used. In addition, subgroup analysis for saline and ATs was performed.

## 5. Conclusions

Treatment with HA eye drops alone, compared with non-HA-based ophthalmic solutions, may reduce dry eye signs and symptoms in patients with DES. Heterogeneity was eliminated through subgroup analysis. Therefore, our results provide insights for clinicians in clinical practice. Further research, in the form of RCTs with large sample sizes, is needed to determine the effectiveness of HA compared with non-HA-based eye drops for treating DES.

## Figures and Tables

**Table 1:** Baseline characteristics of included studies.

First Author	Location	Publication Type	Study Design	Masking	Patients	Follow Up Duration (Days)	Sample Size (N)		Mean Age (Years)	Sex Ratio (M:W)	HA Conc. (%)	Type of Non-HA Eye Drops
							HA	Non-HA				
Groβ [28]	France	Journal article	RCT (Parallel)	Single	Dry eye disease (Moderate)	84	41	39	55.8	24:56	0.1	0.5% CMC
Essa [27]	United Kingdom	Journal article	RCT (Crossover)	Single	Dry eye disease	28	50	50	60.8	35:15	0.15, 0.4	Pospholipid liposome
												0.25% CMC
Pinto-Fraga [11]	Spain	Journal article	RCT (Crossover)	Double	Dry eye patients (Mild)	30	16	16	58.0	8:8	0.2	0.9% Saline
Lopez-de la Rosa [26]	Spain	Journal article	RCT (Crossover)	Double	Dry eye disease (Moderate to severe)	30	16	16	57.5	4:12	0.3	0.9% Saline
Lambiase [19]	Italia	Journal article	RCT (Parallel)	Double	Dry eye patients	14	20	15	56.9	3:36	0.18	Lubricin
Robert [25]	France	Journal article	RCT (Parallel)	Single (Investigator)	Dry eye patients (Moderate to severe)	90	41	44	62.6	16:69	0.18	Hypotonic CE
Kinoshita [20]	Japan	Journal article	RCT (Parallel)	Quadruple	Dry eye patients	28	95	93	55.6	25:163	0.1	2% Rebamipide
Baudouin [29]	France	Journal article	RCT (Parallel)	Single (Investigator)	Dry eye patients	35	29	37	56.8	8:69	0.18	0.5% CMC
Baeyens [12]	France	Journal article	RCT (Parallel)	Double	Dry eye patients (Moderate)	84	100	96	59.3	41:245	0.18	Saline
								91				0.3% Carbomer
Lee [33]	Korea	Journal article	RCT (Parallel)	Single (Observer)	Dry eye patients (Mild to moderate)	56	32	33	38	6:59	0.1	0.5% CMC
Sanchez [16]	Spain	Journal article	RCT (Parallel)	Single (Observer)	Dry eye syndrome or Sjogren’s syndrome	30	15 *	14 *	71.8	All female	0.15	0.5% Carmellose
NCT00938704 [34]	German	Clinical trial	RCT (Parallel)	Double	Dry eye patients	14	37	33	51.5 †	19:51	0.18	0.5% CMC
Rolando [18]	Italia	Journal article	RCT (Parallel)	Open label	Dry eye syndrome	90	9	11	60.3	10:20	0.2	0.5% TSP
								10				1% TSP
Brignole [30]	France	Journal article	RCT (Parallel)	Single (Observer)	Dry eye syndrome (Moderate)	56	10	11	63	1:20	0.18	1% CMC
Condon [31]	United Kingdom	Journal article	RCT (Crossover)	Double	Dry eye syndrome (Severe)	28	34	36	61	12:58	0.1	0.9% Saline
Nelson [35]	United States of America	Journal article	RCT (Parallel)	Double	Dry eye syndrome (Moderately severe)	56	20	15	58.55	4:31	0.1	1.4% PVA
Laflamme [32]	Canada	Journal article	RCT (Crossover)	No comment	Dry eye patients (Severe)	56	12	12	58	Not reported	0.1	1.4% PVA

HA: Hyaluronic acid; N: Number; M: Men; W: Women; Conc.: Concentration; RCT: Randomized controlled trial; KCS: Keratoconjunctivitis sicca; CMC: Carboxymethylcellulose; CE: Cathoic emulsion; TSP: Tamarind seed polysaccharide; PVA: Polyvinyl Alcohol. * number of eyes; † median age.

**Table 2:** Risk of bias assessment for included studies.

First Author	Random Sequence Generation (Selection Bias)	Allocation Concealment (Selection Bias)	Blinding of Participants and Personnel (Performance Bias)	Blinding of Outcome Assessment (Detection Bias)	Incomplete Outcome Data (Attrition Bias)	Selective Reporting (Reporting Bias)	Other Bias
Groβ [28]	?	?	●	?	●	●	●
Essa [27]	?	?	●	●	●	●	●
Pinto-Fraga [11]	?	?	●	●	●	●	●
Lopez-de la Rosa [26]	?	?	●	●	●	●	●
Lambiase [19]	●	?	●	●	●	●	●
Robert [25]	●	●	●	●	●	●	●
Kinoshita [20]	●	●	○	●	●	●	●
Baudouin [29]	●	?	○	●	●	●	●
Baeyens [12]	?	?	●	●	●	●	●
Lee [33]	?	?	○	●	●	●	●
Sanchez [16]	●	?	○	●	●	●	?
NCT00938704 [34]	?	?	●	●	●	●	●
Rolando [18]	?	?	○	○	●	●	●
Brignole [30]	●	?	○	●	●	●	●
Condon [31]	●	●	●	●	●	●	●
Nelson [35]	?	?	●	●	●	●	?
Laflamme [32]	?	?	?	?	●	●	●

●: High risk; ○: Low risk; ?: Unclear risk.

## Data Availability

All data used and referred can be found in PubMed or other sources according to reference list.

## Supplementary Materials

The following are available online at https://www.mdpi.com/1660-4601/18/5/2383/s1, Figure S1: Tear break-up time value in hyaluronic acid (HA) group and non-HA group after excluding the source of publication bias. Non-HA eye drops were classified according to the component: (A) Saline and (B) Artificial tears. SMD: Standardized Mean Difference; CI: Confidence Interval, Figure S2: Ocular surface disease index value in hyaluronic acid and saline groups using random effect model. SMD: Standardized Mean Difference; CI: Confidence interval.
