# Supplementary material for: A Meta-Analysis of the Efficacy of Hyaluronic Acid Eye Drops for the Treatment of Dry Eye Syndrome
Source: Int J Environ Res Public Health. 2021 Mar 1;18(5):2383. doi: 10.3390/ijerph18052383 (PMC7967738; doi:10.3390/ijerph18052383)
Supplement: Supplementary file 1 [file ijerph-18-02383-s001.zip › figures_supple_수정.pptx]

## Slide 1
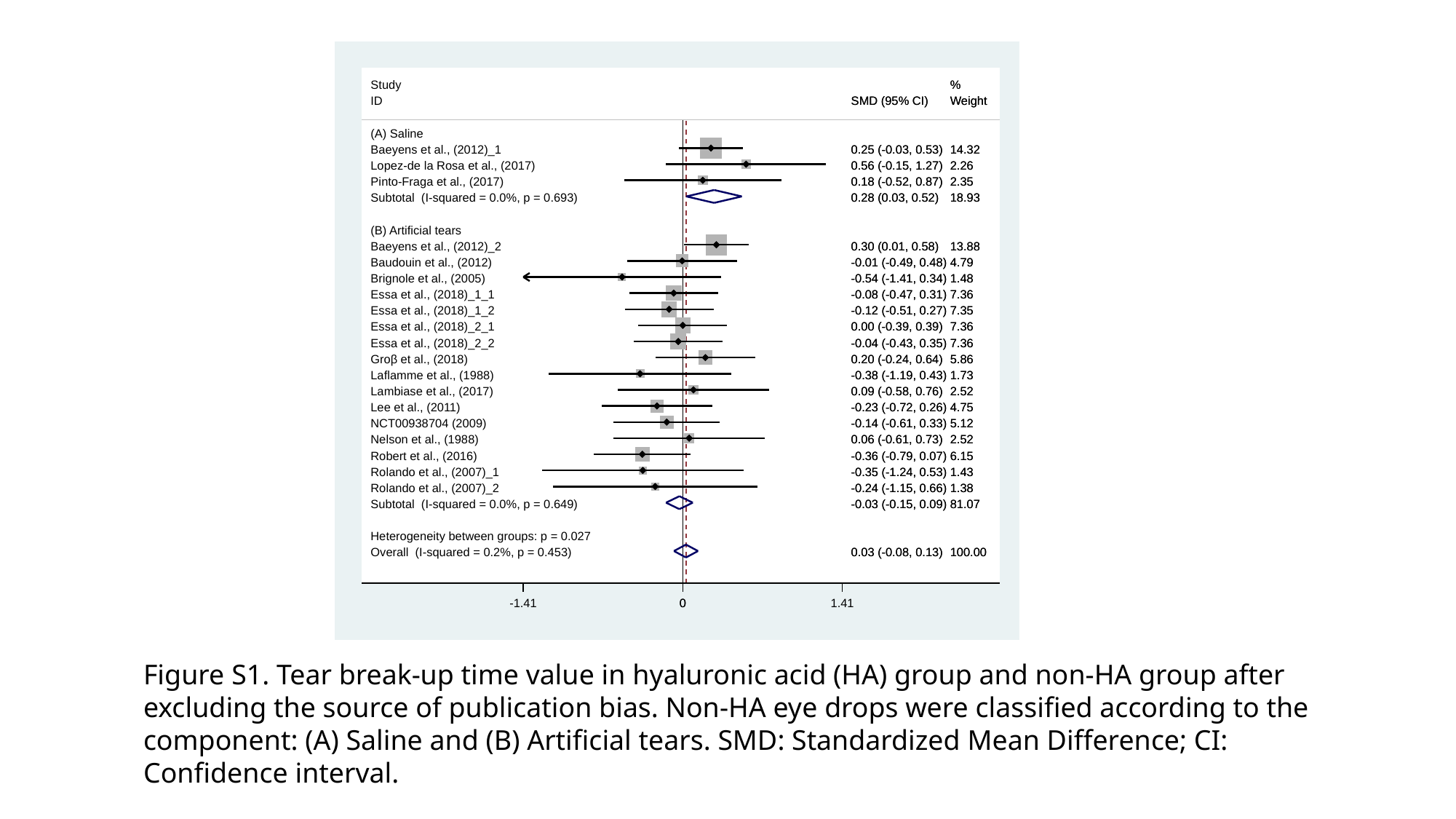

Figure S1. Tear break-up time value in hyaluronic acid (HA) group and non-HA group after excluding the source of publication bias. Non-HA eye drops were classified according to the component: (A) Saline and (B) Artificial tears. SMD: Standardized Mean Difference; CI: Confidence interval.

## Slide 2
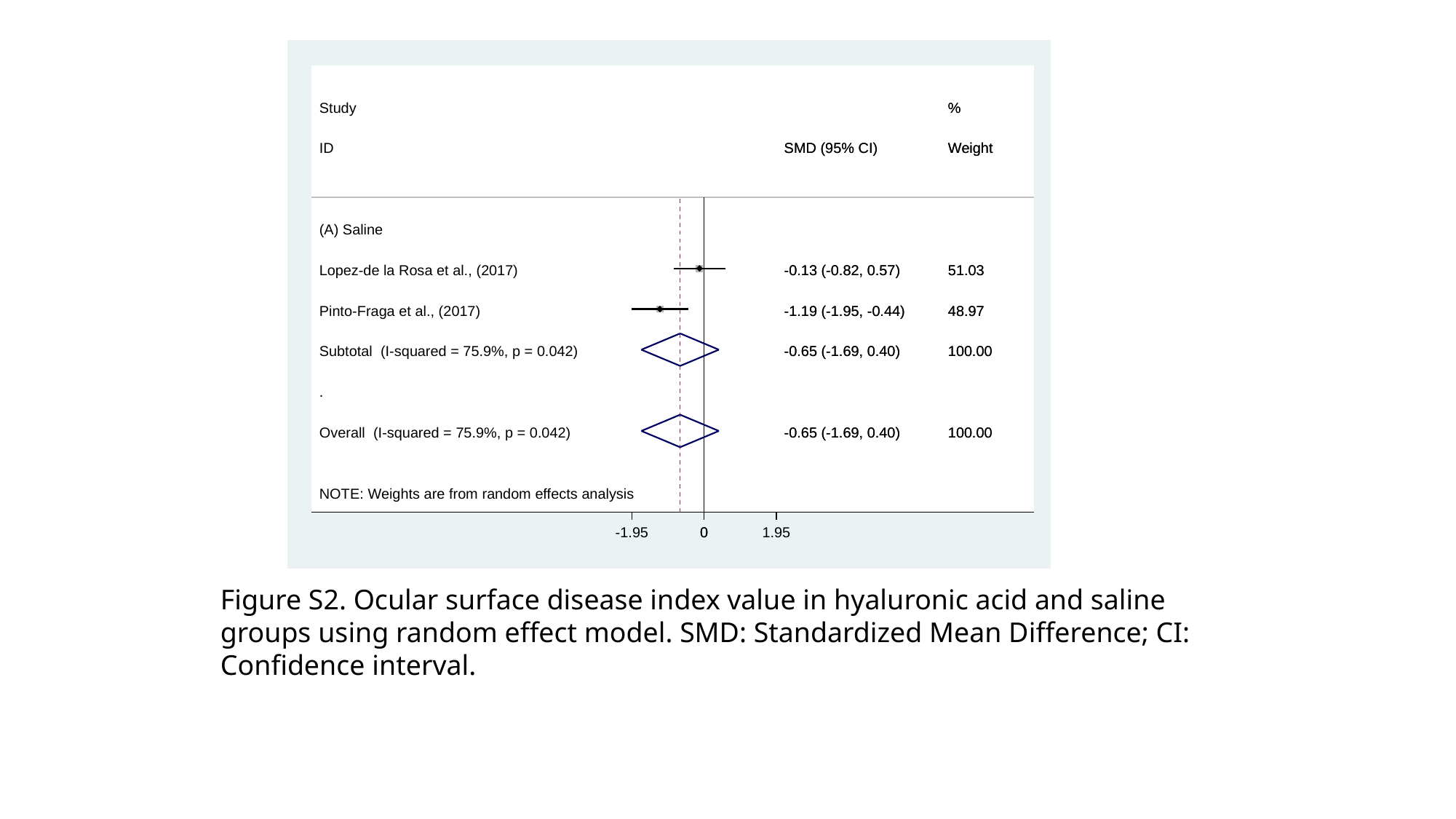

Figure S2. Ocular surface disease index value in hyaluronic acid and saline groups using random effect model. SMD: Standardized Mean Difference; CI: Confidence interval.
